# Design and development of an in-house multiplex RT-PCR assay for simultaneous detection of HIV-1 and HCV in plasma samples

**Published:** 2012-03

**Authors:** M Paryan, Moghadam M Forouzandeh, V Kia, S Mohammadi-Yeganeh, Raz A Abbasali, Samiee S Mirab

**Affiliations:** 1 Biotechnology Research Center, Pasteur Institute of Iran, Tehran, Iran; 2 Department of Medical Biotechnology, Tarbiat Modares University, Tehran, Iran; 3 Food and Drug Laboratory Research Center, Ministry of Health and Medical Education, Tehran, Iran

**Keywords:** HIV-1, HCV, Multiplex RT-PCR, Co-infection

## Abstract

**Background and Objectives:**

HIV-1 and HCV infections are life threatening problems in patients who receive blood products. Serological methods have proven useful in detecting these infections, but there are setbacks that make it challenging to detect these infectious agents. By the advent of Nucleic Acid Testing (NAT) methods, especially in multiplex format, more precise detection is possible.

**Materials and Methods:**

We have developed a multiplex RT-PCR assay for simultaneous detection of HIV-1 and HCV. Primers were designed for highly conserved region of genome of each virus. Using these primers and standard plasmids, we determined the limit of detection, clinical and analytical specificity and sensitivity of the assay. Monoplex and multiplex RT-PCR were performed.

**Results:**

Analytical sensitivity was considered to be 100 and 200 copies/ml for HIV-1 and HCV, respectively. High concentration of one virus had no significant effect on the detection of the other one with low concentration. By analysis of 40 samples, clinical sensitivity of the assay was determined to be 97.5%. Using different viral and human genome samples, the specificity of the assay was evaluated to be 100%.

**Conclusions:**

The aim of this study was to develop a reliable, rapid and cost effective method to detect HIV-1 and HCV simultaneously. Results showed that this simple and rapid method is perfectly capable of detecting two viruses in clinical samples.

## INTRODUCTION

Infection with HIV-1 and HCV, especially co-infection, is a life threatening problem in patients who receive blood products. Therefore, it is absolutely necessary to be certain about the safety of donated bloods and blood products. In the United States and Europe, co-infection with these two viruses is relatively prevalent and the incidence rate is 10% and 25%, respectively (1, 2).

HCV infection in HIV-1 infected patients is usually caused by drug injection (90%) and blood or blood products transfusion (70%) (3, 4). The major cause of death in HIV-1 infected patients who receive HAART which prevents the progress of HIV-1 infection is the HCV mediated hepatotoxicity (5, 6). In addition, simultaneous HIV-1 infection and HCV-related hepatic diseases, like cirrhosis and hepatocarcinoma, rapidly increases in these patients (7, 8).

In addition to co-infection, these viruses are most noticeable infectious agents transmitted by blood. One of the most considerable solutions to overcome the problem is serological tests for screening the antibodies produced against HIV-1 and HCV (9, 10). Serological methods have been used for years to detect viral infections. Although culturing of viruses and direct examination are emphasized, serological methods have proven to be useful in detecting these infections (11).

HIV-1 and HCV could be transmitted in window period in which the infection is not detectable with serological tests (12). Other reasons that disqualify serological tests are antigenic variations of viruses, infections with different serotypes of a virus, presence of silent carriers and lack of antibody in the beginning of the infection. Another disadvantage of these methods is the presence of maternal antibodies which make it impossible to detect infection in new borns from mothers who are infected with HIV-1 or HCV. Furthermore, by means of serological methods, distinguishing patients with active infection from those who completely cured (and they have anti-HCV IgG) is not possible (13–15).

To overcome these setbacks, nucleic acid based testing (NAT) methods for detecting the viral genome have been developed. One of the Advantages of NAT methods in comparison with serological methods includes direct examination of the infectious agent's genome with high specificity and sensitivity. Although the possibility and functionality of NAT have been proven, large scale exploitation of it is limited because of high cost (16, 17). Moreover, pooling of samples employed by several molecular approaches determines a relatively high rate of false-negative results and loss of sensitivity when a low-positive sample is diluted into the pool. To conquer these problems, Multiplex NAT has been developed to make it possible to simultaneously analyze a single sample for multiple infections. So the time and cost needed for NAT assays are significantly reduced (18–21). By the advent of Multiplex RT-PCR, it is now possible to detect simultaneous viral infections with high precision and accuracy. Recently this simple, rapid and cost-effective method has been used to detect bacterial, protozoan and other infectious agents (21–27).

## MATERIALS AND METHODS

### Clinical samples

Eighty different plasma samples were entered in this study. These included 30 samples co-infected with HIV and HCV, 10 HIV positive and HCV negative, 20 HCV positive and HIV-1 negative and 20 HIV-1 and HCV negative samples from healthy individuals. The co-infected and HIV positive samples were obtained from patients of Imam Khomeyni hospital, whose infections had been confirmed by ELISA, Western blot and RT-PCR. HCV positive and healthy samples were obtained from Day hospital, and were confirmed by ElectroChemiLuminescense (ECL) and RT-PCR. All of the samples were transferred to our laboratory considering the biosafety regulations.

### Primer *design*

Primers were designed based on conserved regions of genome for each virus so they were able to recognize all the genotypes of each virus. Totally, 471 sequences of HCV viruses and 1242 sequences of HIV-1 viruses were retrieved from GenBank, NCBI (http://www.ncbi.nlm.nih.gov) and were aligned using MEGA-4 software to identify the conserved regions. *pol* gene of HIV-1 and 5′NCR of HCV genome were selected (Table 1). The length of amplicons for HIV-1 and HCV were 179 bp (nucleotides 4354 to4533) and 241 bp (nucleotide 84 to325), respectively. Moreover, Oligo 6 and AlleleID 6 softwares were used to inspect the compatibility of primer pairs.

**Table 1:** Primer sequences for HIV-1 and HCV.

Name	Sequence	location
HIV-F	5′ GTA CAG TGC AGG GGA AAG 3′	4354
HIV-R	5′ AAC CAG AGI AG[C/T] TTT GCT G3′	4533
HCV-F	5′ CAT GGC GTT AGT ATG AGT G 3′	84
HCV-R	5′ AA AAC TAT CAG GCA GTA CCA CAA G 3′	325

Sequences of each virus were retrieved from GenBank, NCBI and were aligned to locate highly conserved regions. *pol* regions of HIV-1 and 5′NCR of HCV were chosen for primer designing. HIV-F: HIV-1 forward primer, HIV-R: HIV-1 reverse prime HCV-F: HCV forward primer, HCV-R: HCV reverse primer.

### Viral RNA extraction

Blood samples were collected in tubes containing EDTA and were centrifuged at 2500 RPM for 20 minutes. The plasmas were stored at −80°C until use.

To extract viral genomes from 200 µl of plasma samples, High Pure Viral RNA Kit (Roche Diagnostics, Germany) were used according to manufacturer's instructions.

### Reverse Transcription

Viral RNA were reverse transcribed using M-MuLV enzyme (Fermentas, Germany). 1 µl of HIV-R primer (2 µM) and 1 µl of HCV-R primer (2 µM) were added to 5 µl of extracted RNA samples of HIV-1 and HCV in two different 0.2 ml tubes, respectively. RNase and DNase free distilled water were added to a final volume of 12 µl. Then tubes were incubated for 10 minutes in 70°C. Finally, 5 µl enzyme buffer, 2 µl dNTP (10 mM), 0.5 µl RNase Inhibitor (20 U/ µl) and 1 µl M-MuLV RT (200U/ µl) were added and then tubes were incubated at 42°C for 60 minutes. To stop the reaction, tubes were incubated at 70°C for 10 minutes.

To eliminate residual RNA, reverse transcribed cDNAs were treated with RNase H for 20 minutes at 37°C. cDNAs were store at −80°C until use.

### Cloning of cDNAs in order to construct standard Plasmid

Cloning was carried out using Fermentas T/A cloning kit K1213. The cDNAs from genotype 3a of HCV and genotype M of HIV-1 were cloned in PTZ 57R/T according to manufacturer's instructions. Then *Escherichia coli* DH5α strain was transformed chemically according to the instructions mentioned in Sambrook molecular cloning. Plasmids were extracted from bacteria using Qiaprep^®^ Spin Miniprep Kit (Qiagen, Germany). Then plasmids were treated by *Eco*RI restriction enzyme to obtain linear plasmid to use as standards. By running the plasmid on 0.8% agarose gel the quality of plasmids were assessed. Then the concentrations of plasmids were determined using a Nanodrop, and standards were made based on copy number/ml. 10-fold serial dilutions were constructed from 10^6^ to 10^2^ copies/ml.

### Performing RT-PCR using designed primer

In order to amplify the target sequence of each virus, RT-PCR was carried out for each virus cDNA, separately and products were run on 2% agarose gel. 179 bp and241 bp bands indicated the amplification of HIV-1 and HCV target sequences, respectively.

### Detection of HIV-1 and HCV genomes using multiplex RT-PCR

To assess the assay, multiplex PCR was performed in 25 µl PCR reaction using Multiplex PCR kit (Qiagen, Germany). 12.5 µl of 2× Multiplex PCR Master Mix (Qiagen, Germany), 0.4 µl of 10 mM dNTPs mix, 0.4 µl of Taq DNA polymerase (5 U/ µl), 1 µl of 25 mM MgCl2, 0.5 µM of HIV-1 and HCV oligonucleotide primers and 5 µl cDNA were added to the master mix according to manufacturer's instruction. Multiplex PCR were carried out according to the following temperature profile: 95°C for 5 minutes, “40 cycles of 95°C for 20 seconds, 58°C for 30 seconds and 72°C for 35 seconds” and a final extension for 5 minutes. The PCR reactions were performed using ABI 2720 thermocycler. The PCR products were run on 3% agarose gel.

### Sensitivity and specificity

To determine the diagnostic specificity, ten samples of human genome and some blood transmitted viruses were analyzed, as well as performing BLAST using NCBI BLAST program. To determine the clinical specificity, 20 negative samples, were used. These samples were tested previously using serological and molecular methods and confirmed that they were HIV-1 and HCV negative. To determine the analytical sensitivity, the plasmids mentioned above were used. A serial dilution of each standard plasmid from 10^6^–10^2^ copies/ ml was prepared. Multiplex-PCR was performed on each standard in a quadruplicate mode and repeated the next day to inspect the limit of detection (LOD) of the assay.

Finally to assess the clinical sensitivity, 40 positive samples including 30 samples from co-infected HIV-1 and HCV patients and ten manually spiked samples, prepared from mixing plasma of patients with HIV-1 or HCV infection with plasma of healthy individuals, were tested.

## RESULTS

### Performing multiplex RT-PCR and running 3% agarose gel electrophoresis

**Fig. 1:**
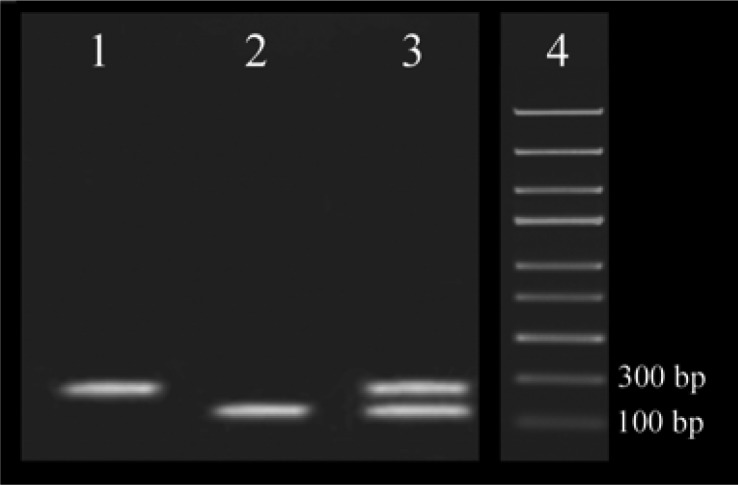
Agarose gel electrophoresis analysis of HIV-1 and HCV monoplex and multiplex amplification. Lane 1 and 2 are the monoplex reactions and lane 3 is the multiplex reaction. HIV-1-specific amplicon is 197bp and HCV-specific amplicon is 241bp. Lane 4 is 100 Kb DNA marker.

Fig. 1 Shows the gel electrophoresis related to Multiplex RT-PCR in which both specific targets have been amplified and separated based on their size.

### Multiplex RT-PCR sensitivity

In order to determine the analytical sensitivity of the multiplex assay, eight multiplex RT-PCR of each standard plasmid were performed (105 copies/ml, 104 copies/ml, 103 copies/ ml, 200 copies/ ml, 100 copies/ml (Table 2). In each group of reactions, the load of each standard was equal. The analytical sensitivity of the assay was 100 copies/ml and 200 copies/ml for HIV-1 and HCV, respectively.

**Table 2:** *Analytical sensitivity analysis.

HIV-1 (Copies/ml)	HCV (Copies/ml)	positive HIV-1	positive HCV
100000	100000	8/8	8/8
10000	10000	8/8	8/8
1000	1000	8/8	8/8
200	200	8/8	8/8
100	100	8/8	6/8

* Eight multiplex reactions in which the concentration of each viral plasmid standard was equal. All eight reactions in each group of standards showed positive results for HIV-1. For HCV, 6 out of 8 reactions were positive for 102 standard. In other groups of standards all eight reactions were positive for HCV. Therefore the analytical sensitivity of the assay considered 100 and 200 copies/ml for HIV-1 and HCV, respectively.

Since the analytical sensitivity of multiplex PCR could be affected by high concentration of each target sequence, a serial dilution of each viral standard were tested against a high copy number/ml [10^6^] standard of the other one. As shown in the Table 3 high viral load of one target sequence does not influence the analytical sensitivity of the other virus with low concentration.

**Table 3:** *Analytical sensitivity of the Multiplex assay with different standard concentration****.

HIV-1 [Copies/ml]	HCV [Copies/ml]	HIV-1 and HCV positive
10^6^	10^5^	8/8
10^6^	10^4^	8/8
10^6^	10^3^	8/8
10^6^	200	8/8
10^6^	100	5/8
10^5^	10^6^	8/8
10^4^	10^6^	8/8
10^3^	10^6^	8/8
200	10^6^	8/8
100	10^6^	8/8

* This experiment was performed to inspect the effect of high concentration of one virus on the amplification of the other one. High concentration of one target has no significant effect on the amplification of the other target with low concentration.

To inspect the clinical sensitivity, 40 co-infected samples were analyzed. Ten samples were manually prepared by adding HIV-1 and HCV infected plasma within healthy plasma. It should be noted that in those ten manually prepared samples, the load of each infected sample was one log higher than the analytical sensitivity of the assay, in order to assess the capability of the assay when the viral load is low.

**Fig. 2:**
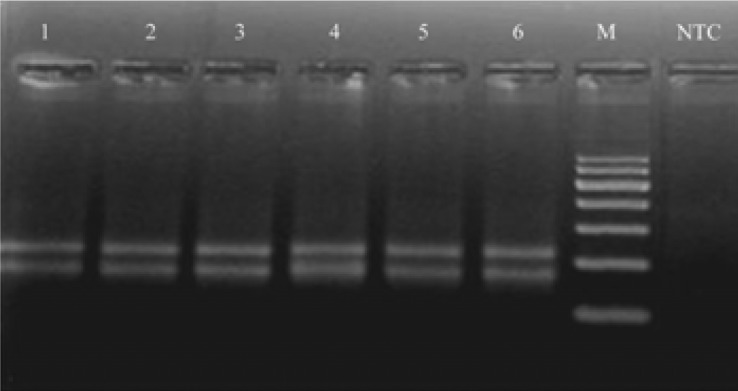
Determining the clinical sensitivity using co-infected samples. The agarose gel above shows part of the results. Lanes 1 through 6 are manually prepared samples by adding HIV-1 and HCV infected plasma together with healthy plasma. M: 100 bp DNA marker, NTC: No Template Control.

Using the designed assay, 39 out of 40 samples were positive for both viruses and one sample was positive only for HCV (Fig. 2). Therefore the clinical sensitivity of this assay was 97.5%.

### Determining the specificity

In order to determine the analytical specificity, first, a BLAST was carried out using NCBI Nucleotide BLAST software. It was proved that the primers do not attach to any other sequences except HIV-1 or HCV. Then, ten different samples including human genome and some blood transmitted viruses, like HBV, HTLV-1, TTV, B19, HSV-1, HSV-2, HHV-6, HHV-7, HCMV and EBV were examined. Only HIV-1 and HCV and no other irrelevant genomes were detected (data not shown).

Clinical specificity was assessed by using 25 negative samples which were proved to be negative by standard serological and molecular methods previously mentioned. No positive results were observed, thus the clinical specificity of the assay considered 100%.

## DISCUSSION

The objective of developing this multiplex RT-PCR was the simultaneous detection of HIV-1 and HCV in patients’ plasma. To do so, conserved regions of genomes of each virus were amplified using specific primer pairs. The concentration of MgCl_2_ and primers were optimized, as these are fundamental factors affecting specificity and sensitivity. Analysis of the multiplex RT-PCR products is based on 3% agarose gel electrophoresis which shows the amplification of the templates. Gel electrophoresis showed two different bands of 179 and 241 bp indicating the amplification of HIV-1 and HCV, respectively (Fig. 1). The first multiplex assay for the detection of HIV-1 and HCV was based on monoplex and simultaneously flowcytometric detection which was cumbersome (28). Other multiplex methods like AMPLINAT PMX test (16) and TMA-based assay (29) and multiplex real-time PCR (18) are sensitive methods for the detection of HIV-1 and HCV, but these methods are expensive and could not be used as a screening method. In contrast, multiplex RT-PCR has considerable advantages, such as being less time consuming because of multiplexing, high sensitivity, reproducibility and straightforwardness.

Candiotti et al. (2004) developed a multiplex real-time PCR for the simultaneous detection of HBV, HCV and HIV-1 based on Hydrolysis Probes (19). Although this method has high sensitivity, it is much more expensive than Multiplex RT-PCR which makes it less optimal as a simple screening method.

In another experiment, De Cringes et al. (2010) used a SYBR Green I Multiplex Real-Time PCR which was able to differentiate HIV-1 and HCV in DBS1 samples based on amplicon melting temperature (30). The analytical sensitivity of the assay was 400 copies/ml for HIV-1 and 2500 copies/ml for HCV which was not highly sensitive. In an article published by Giblini in 2006, it was shown that by using SYBR Green I multiplex real-Time PCR, it is possible to detect the co-infection of HIV-1 and HCV in plasma samples with the sensitivity of 500 copies/ml. The primers were designed for gag region of HIV-1 and 5'UTR of HCV (3). Tang et al. developed a new detection toolvisual DNA microarray for simultaneous and specific detection of human immunodeficiency virus type-1 and hepatitis C virus. The sensitivity of visual DNA microarray was (103copies/ml) (31).

In this article we describe the development of a Multiplex RT-PCR assay for simultaneous detection of HIV-1 and HCV viruses in plasma samples. This assay is able to detect and differentiate these viruses based on the difference in amplicon size on 3% agarose gel with a high sensitivity. In order to design primers, a multiple alignment was performed and the most conserved regions were selected. Based on the BLAST results and inspection of other interfering factors, like DNA extracted from human samples and viruses transmitted by blood, it was shown that the designed primers, and in other words the assay is capable of detecting HIV-1 and HCV and there is no cross reaction with other viruses. The analytical sensitivity of the assay for HIV-1 and HCV was 100 and 200 copies/ml, respectively. As shown in (Table 3) although the presence of high concentration of either virus slightly lowers the sensitivity of the multiplex assay (especially in low concentrations of HCV), it has no effect on analytical sensitivity of the assay. In order to determine the analytical sensitivity, samples from HIV-1 and HCV infected patients with known copy number were used. In order to reduce the error in determining the initial copy number of samples, a mean of three quantifications in three different days using standard commercial kits were calculated and used. It should be noted that in this process, the coefficient of variation was lower than 5% by analyzing 40 HIV-1 and HCV positive samples and 20 samples from healthy individuals. The clinical sensitivity and specificity of the assay were determined to be 97.5% and 100%, respectively. A considerable matter in calculating the clinical sensitivity is that at least, 20 samples out of 40 positive co-infected ones, have low copy numbers (maximally one log more than the analytical sensitivity) of either virus. And this shows the capability of the developed assay to detect low copy number of virus in patients’ plasma; which is crucial in HIV-1 HCV patients.

In addition to high sensitivity and specificity, low cost and simplicity are the major advantages of this assay. Furthermore, the assay is rapid and practical for screening of HIV-1 and HCV in patient plasma and could be exploited in blood screening at blood transfusion organizations.
